# Electrophysiological Responses in the Human S3 Nerve During Sacral Neuromodulation for Fecal Incontinence

**DOI:** 10.3389/fnins.2021.712168

**Published:** 2021-10-11

**Authors:** Gerrit E. Gmel, Paul F. Vollebregt, Marjolein E. G. Thijssen, Rosana Santos Escapa, Eleanor McAlees, Dave Mugan, John L. Parker, Charles H. Knowles

**Affiliations:** ^1^Saluda Medical Pty Ltd, Artarmon, NSW, Australia; ^2^National Bowel Research Centre, Centre for Neuroscience, Surgery and Trauma, Blizard Institute, Barts and The London School of Medicine and Dentistry, Queen Mary University of London, London, United Kingdom

**Keywords:** sacral neuromodulation, fecal incontinence (FI), electrophysiology, evoked compound action potential (ECAP), myoelectric activity, sacral nerve

## Abstract

Intra-operative electrode placement for sacral neuromodulation (SNM) relies on visual observation of motor contractions alone, lacking complete information on neural activation from stimulation. This study aimed to determine whether electrophysiological responses can be recorded directly from the S3 sacral nerve during therapeutic SNM in patients with fecal incontinence, and to characterize such responses in order to better understand the mechanism of action (MOA) and whether stimulation is subject to changes in posture. Eleven patients undergoing SNM were prospectively recruited. A bespoke stimulating and recording system was connected (both intraoperatively and postoperatively) to externalized SNM leads, and electrophysiological responses to monopolar current sweeps on each electrode were recorded and analyzed. The nature and thresholds of muscle contractions (intraoperatively) and patient-reported stimulation perception were recorded. We identified both neural responses (evoked compound action potentials) as well as myoelectric responses (far-field potentials from muscle activation). We identified large myelinated fibers (conduction velocity: 36–60 m/s) in 5/11 patients, correlating with patient-reported stimulation perception, and smaller myelinated fibers (conduction velocity <15 m/s) in 4/11 patients (not associated with any sensation). Myoelectric responses (observed in 7/11 patients) were attributed to pelvic floor and/or anal sphincter contraction. Responses varied with changes in posture. We present the first direct electrophysiological responses recorded from the S3 nerve during ongoing SNM in humans, showing both neural and myoelectric responses. These recordings highlight heterogeneity of neural and myoelectric responses (relevant to understanding MOA of SNM) and confirm that electrode lead position can change with posture.

## Introduction

Sacral Neuromodulation (SNM, also referred to as sacral nerve stimulation), was first established as a therapy for treatment of refractory fecal incontinence (FI) in 1995 (Matzel et al., 1995). The treatment is effective in the long-term [intention-to-treat 54% over a median of 56 months follow up (Thin et al., 2013)], with complete continence achieved in about one third of patients (Altomare et al., 2015). A recent retrospective study showed that efficacy is maintained in 45% of patients at 10 years post-implantation (Desprez et al., 2020). Whilst some evidence has been provided by small double-blind crossover studies, randomized data are lacking, and experimental efficacy has not yet been validated in an adequately powered clinical trial (Thaha et al., 2015; McAlees et al., 2018).

The reasons for treatment failure of SNM are unclear. Sub-optimal lead placement is believed to be one factor, which an international expert group have recently sought to improve by standardization of lead placement (Matzel et al., 2017). Refinement of surgical techniques and intraoperative testing for a motor response both aim to minimize the distance between the electrodes and the sacral nerve. While this ensures that the stimulus reaches some fibers of the sacral nerve, testing for a motor response alone gives a very limited account of the fibers being activated by stimulation. A recent study comparing SNM trial success in patients with overactive bladder, urinary retention and FI found no continuous association between the motor thresholds and trial success (Adelstein et al., 2019). Further, lead migration post-implantation is a common occurrence which requires reprogramming and can lead to loss of efficacy (Ezra et al., 2020).

In this study, we aimed to (1) investigate the nature of evoked neurophysiological responses to stimulation during SNM, separating these into neural responses and myoelectric responses; (2) classify neural responses by activated fiber types; (3) evaluate the effect of patient posture and movement on fiber activation.

## Materials and Methods

### Patient Selection and Study Procedures

Eleven patients (10 women and 1 man) with refractory symptoms of FI [failure of non-surgical treatments according to the United Kingdom NICE standard (Norton et al., 2007)] who had been selected and scheduled for SNM were recruited to take part in the study and gave informed consent to participate. The research protocol was approved by the Research Ethics Committee (REC reference 17/LO/1048). Recordings were taken during standard patient visits in addition to all standard of care procedures:

1. During the first stage procedure to implant the tined lead (“Trial Surgery Visit,” about 5–10 min after placement was completed according to standard practice).
2. Post-lead implantation before the patient left the hospital (“Trial Programming Visit,” for about an hour or as time permitted prior to the patient receiving their external trial stimulator).
3. Pre-second stage procedure to implant the pulse generator (“Trial End Visit,” for about an hour or as time permitted, prior to preparing for the second stage surgery).
4. During the second stage procedure to implant the pulse generator (“Perm Surgery Visit,” about 5–10 min prior to lead removal or permanent stimulator placement. This recording session was only completed if additional recordings were required that could not be obtained in the previous sessions).

**FIGURE 1:**
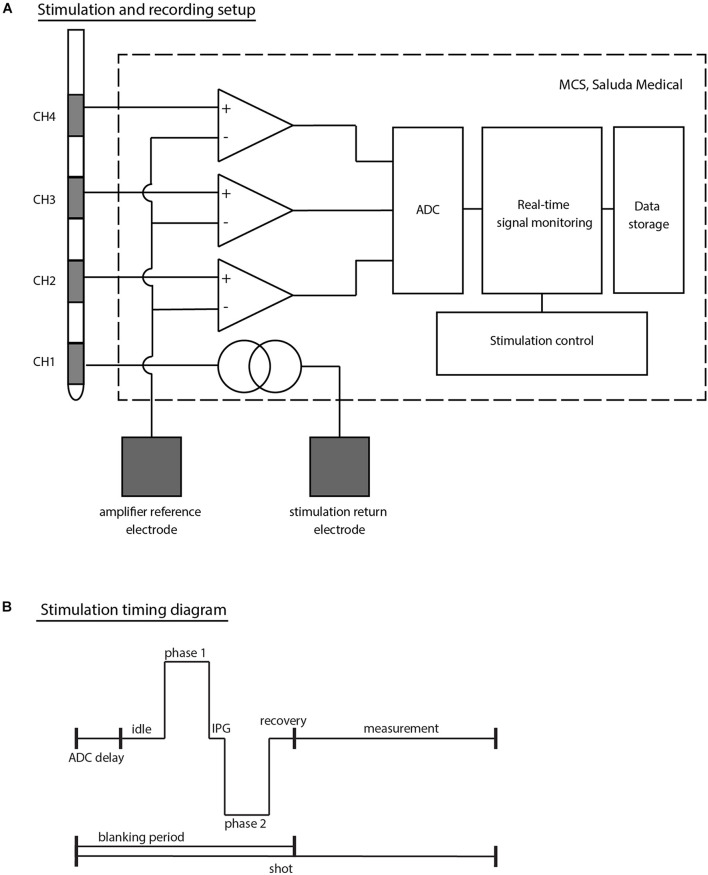
**(A)** Schematic top-level representation of the stimulation and recording setup. The example given corresponds to a setup in which stimulation is applied on CH1 (most distal electrode of a Medtronic 3889 SNS lead) and electrophysiological recordings taken from CH2 to CH4 simultaneously. Both the stimulation return electrode and the amplifier reference channel (common to all amplifiers) are external electrodes (either adhesive carbon rubber pads or subdermal needle electrodes). **(B)** Timing diagram of stimulation outlining the stimulation sequence. Each stimulus and recording sequence is stored in shots. The amplifiers are blanked during the stimulation phase and for short periods before and after stimulation to avoid saturation of the electronics. The MCS design with the choice of ADC leads to a short (753 μs) delay at the beginning of each shot prior to the first stimulus phase. IPG denotes the inter-phase gap in this diagram.

All patients were implanted unilaterally at the S3 level (left or right) with an InterStim^®^ quadripolar electrode (Model No: 3889, Medtronic, Dublin, Ireland, with 3 mm contacts and 3 mm contact spacing) following the standardized technique (Matzel et al., 2017). Throughout this article, CH1 denotes the most distal electrode (near the tip of the lead), and CH4 the most proximal electrode (see Figure 1A).

During the implant procedures, muscle contraction thresholds were recorded (distinguishing between toe contractions and pelvic floor/anal sphincter contractions). Stimulation sensations (intensity, location, and quality) were recorded based on patient feedback during the Trial Programming Visit and Trial End Visit. For this purpose, the stimulus current was slowly ramped up (so called “current sweeps”) and the patient was asked when they first felt the stimulation (threshold level), at which point the stimulation was strong but comfortable (comfort level), and at which point the stimulation became uncomfortable (maximum level).

To assess the effect of movement on electrophysiological responses to stimulation, the patients were asked to perform controlled posture changes during Trial Programming Visit and/or Trial End Visit as long as time permitted and the patient was comfortable performing these tests on the day. During a posture change assessment, the stimulus current was first ramped up to a level at which the stimulation sensation was clear but comfortable with the patient in a sitting position. The patient was then asked to stand up, take a few steps (the distance being limited to a few meters by the length of the cable), and sit back down again. The electrophysiological responses during that time period were analyzed off-line after the session.

### Stimulation and Recording Setup

A custom external stimulator was connected to the proximal end of the Medtronic leads to capture the electrophysiological recordings allowing both real-time monitoring during experiments and further processing offline [the Multi-Channel System Mark II (MCS); Saluda Medical, Artarmon, NSW, Australia]. The MCS comprises custom-made neural amplifiers with 100x gain which record from all non-stimulating electrodes simultaneously (Parker et al., 2013). Responses were filtered with a 4 kHz single-pole anti-aliasing filter and sampled at 30 kHz with a United Electronic Industries data acquisition system (Walpole, MA, United States). The data acquisition unit itself contains an anti-aliasing filter at the Nyquist frequency (15 kHz). While the neural amplifiers are blanked during the stimulus to avoid saturation, recording is enabled after a brief recovery period of 100 μs after the stimulus.

At each patient visit, stimulation was set up in a monopolar configuration with an external ground electrode. A second external electrode (placed at least 10 cm away from the implanted electrodes) was used as a common reference for all neural amplifiers. The external ground and reference electrodes were chosen to be either a ground pad (Model No: 041826, Medtronic, Dublin, Ireland) or a subdermal needle electrode (Model No: 510123-2, TerniMed, Bielefeld, Germany) placed on the patient’s arm or back based on patient comfort and usability (see Figure 1A).

Biphasic stimulation pulses were applied using predominantly the industry standard stimulation parameters [14 Hz stimulation frequency and 210 μs pulse width (PW)]. In some cases, the stimulus frequency and PWs were changed in an attempt to optimize signal detection. In particular, 5 Hz stimulation was used sometimes during the Trial Surgery Visits to help identify motor contractions visually. The PWs were shortened in some experiments to a minimum of 30 μs in order to reduce the amplifier blanking period and allow the recording of signals within a few tens of microseconds after the beginning of the stimulus pulse.

Electrophysiological measures are stored in “shots” containing one stimulus period each (see Figure 1B). Due to idiosyncrasies in the signal acquisition chain of the MCS, each stored shot starts a few hundred microseconds before the stimulus pulse (753 μs). To reduce random noise and improve the signal-to-noise ratio, shots were averaged by either a set number (e.g., shots were averaged 10 by 10), or by stimulus parameter change (e.g., for a current sweep, all shots at a given stimulus current will be averaged together, giving one averaged trace per current).

To clean up the recordings, stimulus artifact was removed by applying an exponential fit to each shot and subtracting the result from the trace. Simple peak detection was used to characterize the components of the electrophysiological responses and their respective amplitudes, no further signal processing or filtering was performed.

### Electrophysiological Response Classification

Recent advances in the field of neuromodulation have made it possible to record electrically evoked electrophysiological responses from nervous tissue during ongoing stimulation less than a millimeter away from the stimulus site (Parker et al., 2012, 2013; Gmel et al., 2015). On electrodes placed near neural tissue, three types of evoked electrophysiological responses have been previously described: propagating neural potentials (evoked compound action potentials, or ECAPs), non-propagating neural potentials (interneuron firing), or myoelectric potentials [the far-field recording of compound muscle action potentials (CMAPs) evoked by activation of motor fibers]. As the sacral nerve does not contain any interneurons, only ECAPs and myoelectric responses were expected to be observed.

#### Myoelectric Responses

Muscle activation can occur either via direct activation of Aα motor neurons, or via a reflex mechanism mediated by proprioceptive fibers. Motor unit action potentials (MUAPs) originate near the middle of the muscle, at the neuromuscular junctions of their Aα motor neurons, and propagate in each direction toward the bones to which the muscle is attached. The combined field generated by all activated motor units is the CMAP which can be observed from electrodes placed in close proximity to the muscle (Beck, 2006).

In the far-field of the muscle (the point at which the size of the muscle is small compared to the distance of the muscle to the electrode), electrodes will pick up the total electrical field of the CMAPs propagating in both directions across the muscle. The resulting signal will therefore last longer than a CMAP recorded at the surface of the muscle and will be seen as non-propagating from the point of view of the recording electrodes (Dumitru and King, 1991; Dumitru and Jewett, 1993). A non-propagating signal observed during SNM in this study was therefore classified to be a myoelectric response, as all muscles innervated by the sacral nerves are in the far-field of the stimulus and recording electrodes.

Although a myoelectric response indicates the presence of a motor response (muscle contraction), the amplitude relationship is not linear and depends on the stimulus frequency and the type of muscle fibers being activated (Sandercock et al., 1985). Therefore, the presence of myoelectric responses indicates that Aα fibers were activated by the electrical stimulation but only gives limited insight on the nature and contractile strength of the motor response.

To investigate which muscle group generates the far-field myoelectric responses, we recorded visual muscle contractions obtained during Trial Surgery Visit current sweeps. As distinguishing between pelvic floor and anal sphincter contractions proved difficult, they are discussed together in this article. Distinction will only be made between sphincter/bellows and toe contractions observed during Trial Surgery Visit tests.

#### Neural Responses

As the lead with its electrodes is placed adjacent to the sacral nerve, neural responses elicited by stimulation will propagate along the axis of the lead, distinguishing them from non-propagating myoelectric responses.

**FIGURE 2:**
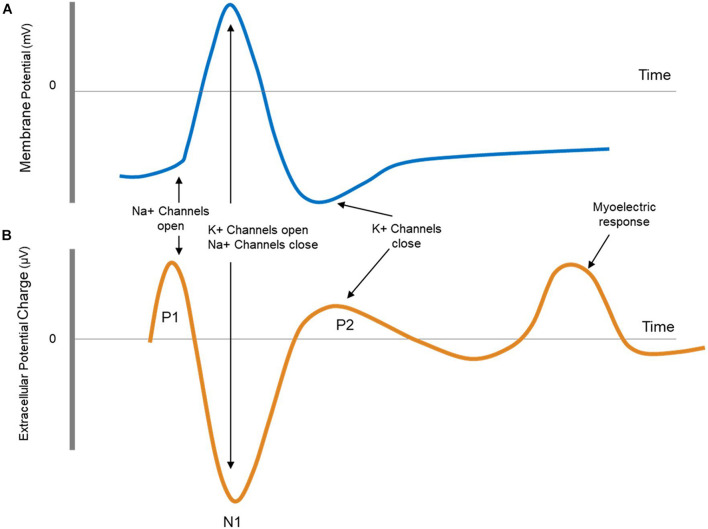
**(A)** Schematic representation of an action potential as measured intracellularly. **(B)** Schematic representation of an evoked compound action potential (ECAP) followed by a myoelectric response. The ECAP is measured extracellularly and is therefore opposite in sign and smaller (μV) compared to the intracellular action potential. The shape of myoelectric response can take a number of forms (multiple phases, varying polarities).

The ECAP is the extracellular measure of the sum of all action potentials (APs) of a population of nerve fibers which have been activated by the stimulus. Being in the near field of the nerve fiber’s response causes a characteristic and predictable triphasic waveform consisting of two positive peaks (P1 and P2) and one negative peak (N1). The first positive peak (P1) is caused by the capacitive coupling through the membrane of the incoming APs to the recording site. The following negative (N1) and positive peaks (P2) are the extracellular counterparts to the membrane potential often described in textbooks when discussing the influx and outflux of sodium and potassium ions that form the AP. Figure 2 shows the shape of a typical ECAP followed by a myoelectric response. The shape of the myoelectric response cannot be predicted with our current understanding, and it can have one or more phases of either negative or positive polarity.

A single-fiber AP elicited by electrical stimulation in the middle of the axon will propagate in both directions away from the stimulus site. Many properties of axons are linked. Most importantly, axons with larger diameters also have thicker myelin sheaths and larger internodal spacing, leading to faster conduction velocities. To a first order, fiber diameter and conduction velocity are linked by a factor of 6 (Waxman, 1980). Conduction velocity measurements are therefore a good surrogate measure for fiber type. Furthermore, information of similar nature is generally transmitted in the nervous system via fibers of the same type. For example, proprioceptive information, required for muscle control, travels in fast fibers, classically labeled as Aα, which have a conduction velocity of around 80–120 m/s. Visceral sensory information on the other hand travels along slower fibers, classically labeled Aδ, which have a conduction velocity of around 5–30 m/s (Purves et al., 2012). Fibers of different types have diameters (and therefore conduction velocities) which follow a normal distribution around their mean. Although some overlap may exist, for all intents and purposes, fiber types can be separated by their conduction velocities (Parker et al., 2017).

Evoked compound action potentials propagate along a bundle of nerves at a conduction velocity determined by the size of the activated fibers. This property can help determine the nature of stimulated fibers from the conduction velocity of the ECAP. As there are more functions than fiber types in the human body, most fiber types have been given more than one name, depending on what type of information they carry (see Table 1). With electrical stimulation of fibers in a mixed nerve, only knowledge of the neuroanatomy can link a given fiber type to a function. In some cases, this might not be possible.

**TABLE 1:** Fiber classification based on conduction velocity used in this publication [compiled from Purves et al. (2012) and Parker et al. (2017)].

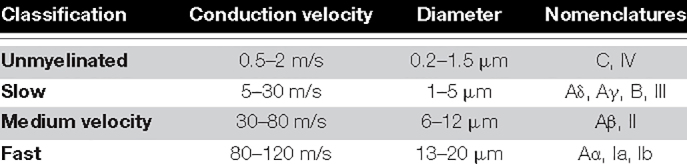

In order to classify the nature of the stimulated fibers during SNM, we obtained the conduction velocities of the N1 peak of the recorded ECAP and classified responses into unmyelinated, slow, medium, and fast, according to Table 1. When the N1 peak of the ECAP was truncated by the blanking period, the P2 peak was used [a description of the blanking period and the stimulus setup has been published previously (Gmel et al., 2015)]. Although Aδ has been separated from Aγ in previous studies (Parker et al., 2017), the classification used in this publication remains conservative allowing for overlap of function in the slow fibers group. The amplitude of each component of the response by the peak-to-peak amplitude of the N1 peak and the following P2 peak for neural responses, or the peak-to-peak amplitude of the largest negative and largest positive peaks of myoelectric responses.

## Results

### Patient Demographics

Eleven patients were recruited [10 female, median age 59 (range 47–76) years at time of testing]. Patients’ clinical characteristics, symptomology and results of anorectal physiologic testing are summarized in Supplementary Table 1. A sensory response to stimulation was reported by all patients except for Patient 3 who presented with symptoms of FI following spinal injury at the L1 level. No study-related adverse events were reported. All patients with the exception of Patient 03 passed their trial and went on to have a permanent implant.

### Electrophysiological Responses

Despite consistent S3 placement for all patients, considerable inter-patient variability was observed in elicited electrophysiological responses. The most common electrophysiological pattern evoked by stimulation was composed of a medium-velocity neural response and a myoelectric response. In a small number of patients (4 out of 11), a slow neural component (with amplitudes of several millivolts) dominated the evoked response. Table 2 lists the electrophysiological responses observed in each patient. Changes in response type between study visits were not observed.

**TABLE 2:** Symptoms and responses observed in each patient.

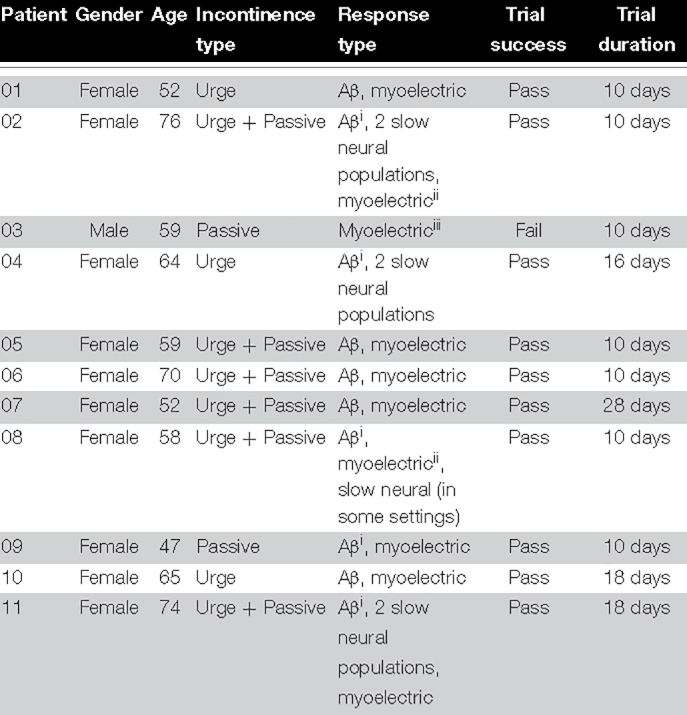

*The “urge” incontinence type designates the presence of urgency episodes with and without involuntary leaks whereas the “passive” incontinence type designates patients which present with involuntary leaks without any sensation.*

*^i^Denotes a deduction of Aβ activation from the reported stimulation sensation although the neural response was masked by artifact or other signals and could not be observed distinctly.*

*^ii^Denotes the presence of a myoelectric response which, due to its overlap with the slow neural response cannot be analyzed separately.*

*^iii^Patient 03 reported no sensation and no Aβ response was observed. The response might have been present but masked by artifact.*

#### Medium Velocity Neural Responses

**FIGURE 3:**
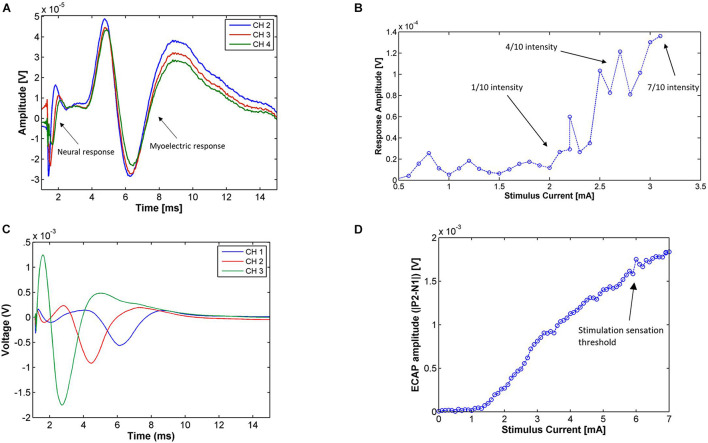
**(A)** Electrophysiological responses from Patient 01 during the Trial Programming Visit from monopolar stimulation on CH1 at the patient’s maximum level (14 Hz, 0.6 mA, 210 μs pulse width, average of 194 shots). The electrophysiological response is composed of a propagating neural response [with a medium conduction velocity (∼45 m/s)] followed by a myoelectric response. **(B)** Amplitude of the medium-velocity neural response [measured as P2-N1 peak-to-peak amplitude of the average trace at each current (minimum 7 shots per stimulus current)] in Patient 06 during the Trial Programming Visit for stimulation on CH1 and recording on CH4 (14 Hz, 30 μs pulse width). The patient reported a very mild stimulation sensation intensity (scored as 1 out 10) at 2.1 mA, a present but comfortable stimulation intensity (scored as 4 out of 10) at 2.8 mA, and a strong but not uncomfortable stimulation sensation (scored as 7 out of 10) at 3.1 mA. **(C)** Responses for stimulation on CH4 of Patient 04 (14 Hz, 6.8 mA, 100 μs pulse width, average of 322 shots) during the Trial Programming Visit. The conduction velocity of this response was approximately 3.5 m/s. **(D)** Amplitude of the slow neural response [measured as the P2-N1 peak-to-peak amplitude of the average trace at each current (minimum 7 shots per stimulus current)] on CH4 for the range of applied stimulus currents in Patient 04. Stimulation was applied on CH2 (14 Hz, 100 μs pulse width). The neural response amplitude rises linearly with the stimulus current after a threshold, however, as opposed to the medium-velocity neural response, the response amplitude does not reflect the intensity of the stimulus sensation. The patient reported a sensation only after the neural response amplitude surpassed 1.5 mV.

Figure 3A shows the neurophysiological responses from Patient 01, composed of a propagating neural response followed by a myoelectric response. The neural response was somewhat masked by the blanking period of the neural amplifiers and the stimulus artifact. Using the N1 peak of the response, the conduction velocity of the neural response was calculated to be approximately 45 m/s, falling into the medium velocity group. Across all patients, conduction velocities of medium-velocity fibers ranged from 36 to 60 m/s with an amplitude at maximum sensation less than 1 mV. A likely candidate for this type of fiber are cutaneous sensory Aβ fibers.

To test whether these medium-velocity responses were indeed cutaneous sensory fibers, we recorded the sensation threshold reported by the patients during the current sweeps and analyzed the neural response amplitude with respect to stimulus current (in so-called recruitment curves). The recruitment curve for Patient 06 is given in Figure 3B. The linear part of the recruitment curve coincided with an increase in the patient’s perceived stimulus sensation. Although stimulation induced a sensation in almost all patients (with the exception of Patient 03) and on almost all electrodes, Aβ responses were often masked by the stimulus artifact. Table 3 therefore only lists the experiments in which an Aβ response was observable. From Table 3 it follows that on average the absolute percentage difference between observed neural response threshold and perception threshold was 14.6% (standard deviation of 10.3%). Along with the conduction velocity range of the observed neural responses, we concluded that these medium-velocity fibers are predominantly Aβ fibers transmitting cutaneous sensory information, and when stimulated, produce a sensation in the corresponding area of innervation (typically the perineum, anus, and/or vagina).

**TABLE 3:** Relation between medium-velocity neural response threshold and sensation threshold for all experiments in which a clear Aβ neural response was observable.

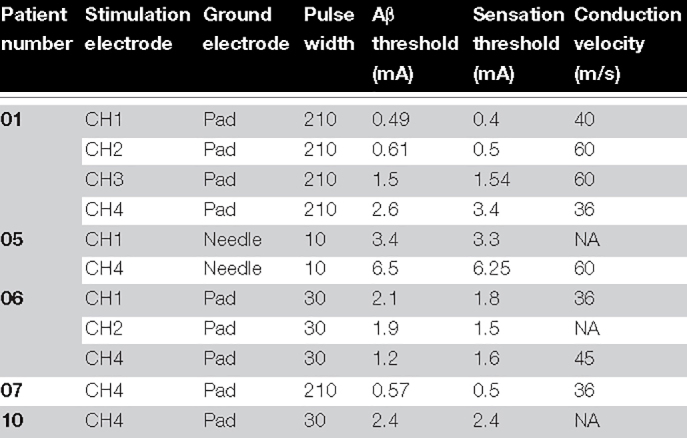

*When the response was only observable on one channel due to low signal-to-noise or signal-to-artifact ratios, the conduction velocity could not be assessed (marked as “NA”).*

#### Myoelectric Responses

Myoelectric responses were typically of the same order of magnitude as the medium-velocity neural responses at maximum stimulation amplitude, although in rare cases they could be measured above 1 mV. In three patients, myoelectric responses plateaued before the patient’s sensory maximum threshold was reached.

Despite the challenges inherent to the methodology, the results demonstrate the link between myoelectric responses and pelvic floor/anal sphincter contractions (see Supplementary Table 2). All patients had some pelvic floor/anal sphincter contraction during the Trial Surgery Visit, however, in four patients, a large slow neural response overlapped the myoelectric response, which could therefore not be analyzed. Big toe flexion did not appear to have a counterpart in electrophysiological responses recorded from implanted SNM electrodes. Patients 05, 07, and 10 all had one or more contacts which gave pelvic floor/anal sphincter responses only and showed a clear myoelectric response. The pelvic floor/anal sphincter response thresholds were on average within 30% of the myoelectric response threshold.

#### Slow Neural Responses

A typical slow neural response is shown in Figure 3C and propagated at a velocity of 3.5 m/s. Across subjects, the conduction velocity of the slow responses varied, but was always less than 20 m/s. Slow neural responses were observed in Patients 2, 4, 8, and 11, sometimes mixed with myoelectric responses due to their overlapping latencies. The observed slow neural responses had properties that separated them substantially from medium-velocity neural responses and the myoelectric responses. They were not found to correspond to a cutaneous sensory response, or any other sensory response reported by the patient. An example of this is given in Figure 3D where the response surpassed 1.5 mV before any sensation was reported by the patient. The amplitude of the slow responses has been found to be up to two orders of magnitude larger than the medium-velocity and myoelectric responses, depending on the stimulation and recording parameters (over 10 mV in some cases).

#### Effect of Posture Changes on Fiber Activation

Posture changes (moving from a sitting position to standing and back) were found to modulate neural and myoelectric responses. Generally, only response amplitude was modulated by movement (either decreasing or increasing the amplitude of the response). However, in Patient 04, movement induced changes in both amplitude and nature of the neural response.

**FIGURE 4:**
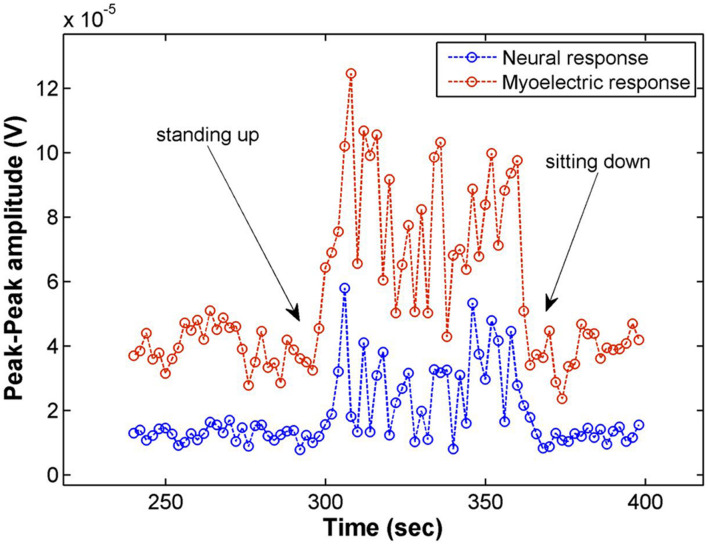
Electrophysiological response amplitudes in Patient 01 recorded on CH4 for stimulation on CH3 (14 Hz, 3.9 mA, 210 μs pulse width), averaging 28 shots for each data point (2 s). Moving from a sitting to a standing posture increased the amplitude of both the myoelectric response as well as the Aβ response. The patient reported a slight increase of the stimulation sensation while standing.

Figure 4 shows the amplitude of the neural and myoelectric components of the response in Patient 01 over the duration of a posture change (going from sitting to standing and back to sitting) with constant stimulus parameters. An increase in amplitude of both the Aβ and the myoelectric responses was observed; the patient reported stronger stimulation sensation while standing, coherent with an increase in Aβ activation. While the myoelectric response moved in tandem with the neural Aβ response in this experiment, this did not generalize over the rest of the posture change assessments performed on other patients. The myoelectric response sometimes moved in tandem, sometimes in reverse to the neural response, and unlike the Aβ, did not correlate with changes in sensation reported by the patient.

**FIGURE 5:**
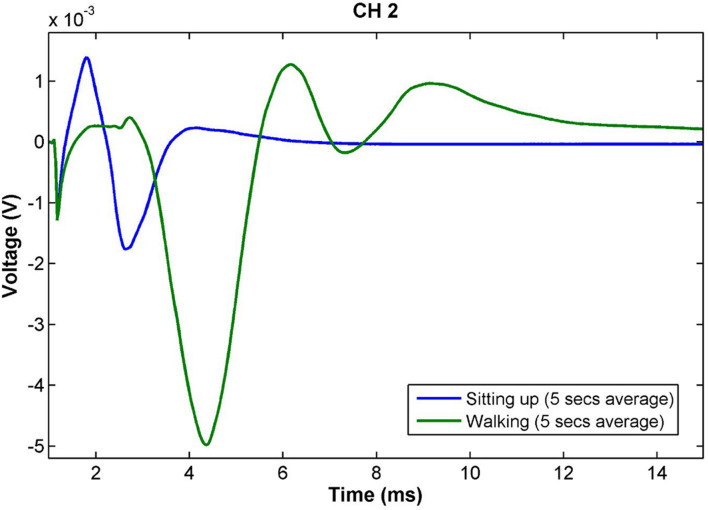
Slow neural responses observed during a posture change in Patient 04 for stimulation on CH2 with constant stimulus parameters (14 Hz, 2.3 mA, 100 μs pulse width). The amplitude of the neural response while walking is more than twice as large as that during sitting. Further, the latency and number of peaks increases, indicating a larger number of different fiber types and lower conduction velocity than in the sitting posture. The patient did not have any stimulation sensation during these measurements. Both traces are the result of averaging the responses obtained over 5 s of continuous recording (70 shots).

Figure 5 shows the neural responses in two postures for Patient 04. In addition to large variations in response amplitude, the latency and number of peaks also changed, indicating that stimulation activated fibers of different nature. Variations of this sort also occurred within the same overall posture and was induced by small changes within it (e.g., leaning back slightly while sitting). The patient reported no sensation from stimulation regardless of the amplitude or morphology of the response.

## Discussion

To our knowledge, this is the first study presenting evoked electrophysiological responses recorded from the human sacral nerve during active stimulation. We recorded neural responses of varying conduction velocities as well as myoelectric responses. These electrophysiological responses showed a large degree of inter-patient variability indicative of fundamental differences in the nature of the fibers activated in each patient. Additionally, the amplitude of evoked responses at a stimulation sensation intensity rated as comfortable by the patient varied by 2–3 orders of magnitude (from several microvolts to several millivolts) depending on the patient and the type of response.

Our results suggest that medium-velocity fibers are sensory Aβ fibers conveying (at least in part) the sensory responses experienced by patients. The amplitudes of the Aβ fiber responses (ranging from a few microvolts to several hundred microvolts at higher stimulus amplitudes), are in line with similar recordings made from epidural spinal cord stimulation electrodes above the dorsal columns, comparable in design to the SNS leads used in this report (Parker et al., 2012; Mekhail et al., 2019; Russo et al., 2020). The amplitude of the slow fiber responses is *a priori* unexpected given that smaller fibers generate smaller action potentials and indicates that a large number of fibers are activated simultaneously. While stimulation amplitude was bounded by patient comfort, itself mediated by the Aβ fiber response, slow fiber activation did not elicit a sensory response in patients. The difference in response amplitude is a reflection of patient perception and comfort rather than of relative number of fibers in the sacral nerve. The patients do not feel the slow fibers and therefore stimulation can activate a majority of them (as demonstrated by the plateauing growth curve), while stimulating Aβ fibers leads to discomfort well before all available fibers have been activated.

The recordings also allowed us to postulate with relative confidence that the myoelectric responses corresponded to pelvic floor/anal sphincter contractions (as opposed to big toe flexion). These results are corroborated by a recent study by Vaganée et al. (2020) investigating low-latency EMG recordings (<10 ms) from intravaginal probes and needle electrodes in the external anal sphincter during SNM. The group found that these low-latency EMGs were associated with the visual motor contractions (pelvic floor) used clinically for intraoperative lead placement (Vaganée et al., 2020). They postulate that these latencies are reflective of direct motor activation from efferent fibers rather than via a mono- or polysynaptic reflex. Monosynaptic reflex muscle responses have been recorded from the spinal cord in humans with latencies of around 10 ms, it is possible a similar pathway exists in the sacral region (Parker et al., 2012; Parker, 2017).

The observed Aβ neural and myoelectric responses are expected from clinical practice (sensations reported by the patient and muscle contraction used for lead placement, respectively). The existence of slow responses was, however, unexpected and the exact nature of these responses could not be determined unequivocally. Their conduction velocity corresponded to both sensory Aδ fibers (visceral sensory fibers) as well as preganglionic parasympathetic B fibers, neither of which would elicit a direct perceptible response that could be reported by the patient. Further, both visceral afferents as well as parasympathetic efferents could contribute to the therapeutic qualities of SNM as they both innervate the target organs. Surprisingly, despite the well-documented role the parasympathetic and sympathetic nervous system play in the storing and voiding of urine and feces, these fibers are often only mentioned indirectly in published attempts to explain the mechanisms of action of SNM. In a review article, Gourcerol et al. (2011) postulate that one of the more likely mechanisms of action of SNM is the activation of sympathetic fibers via a reflex response mediated by somatic afferent activation. They do, however, mention the possibility of the activation of parasympathetic fibers directly as well.

The reason for heterogeneity of observed responses as well as a finer determination of the fiber types could potentially be found in the neuroanatomy of the sacral nerves. In an impressive feat of patience and diligence, Hauck et al. (2009) counted over 200,000 nerves in the human sacral nerve roots in order to determine where to best target slow conducting fibers. They also showed that sacral nerve fibers run in fascicles within the nerves and therefore maintain a certain level of segregation. The location of the lead around the nerve is therefore likely to have a major impact on the fiber types evoked by the stimulation.

Heterogeneity in responses could be one of the main factors that have hindered the elucidation of the mechanisms of action of SNM. As summarized by Carrington et al. (2014), evidence on the effects of SNM on motility, sensation, and contractile force of the colon, rectum and anus remain inconclusive with some reports giving contradicting results. Overall, SNM seems to have both local and central effects ranging from changes in colonic motility to increased sensory evoked potentials in the cortex. Given the heterogeneity of the responses we observed and the wide range of functions associated with the activated nerves, it is no surprise that mechanistic studies have yielded a wide range of results. We postulate that in order to investigate the mechanisms of action of SNM across patients, the evoked fiber types need to be taken into consideration as well as the precise etiologies causing the symptoms.

The large variations in response amplitude observed during simple posture changes highlight a problem known across the field of neuromodulation: as the distance of the electrodes to the tissue changes, even slightly, a constant stimulus amplitude can lead to substantial changes in nerve recruitment. The examples given here show neural responses more than double as the patients changed their posture from sitting to standing. This problem has been well documented in spinal cord stimulation (SCS) applied to the treatment of chronic neuropathic pain, and closed-loop SCS, which automatically adjusts the stimulus amplitude to maintain a constant level of neural recruitment, has been shown to provide significant improvements to open-loop SCS (in which the current is kept constant) in the long term (Mekhail et al., 2019; Brooker et al., 2021).

The combination of intraoperative monitoring of neural responses and the implementation of closed-loop stimulation is likely to lead to substantial improvements in the therapeutic efficacy of SNM. A long-term follow-up study is currently being designed to expand on the findings presented here and will investigate both aspects in a chronic implant study in which the electrophysiological components will be monitored continuously and used for programming and delivering closed-loop SNM.

## Data Availability Statement

The datasets presented in this article are not readily available because the data is the property of Saluda Medical. The data can be made available upon request at the discretion of Saluda Medical. Requests to access the datasets should be directed to GG, gerrit.gmel@saludamedical.com.

## Ethics Statement

The studies involving human participants were reviewed and approved by the London – Harrow Research Ethics Committee (REC reference 17/LO/1048). The patients/participants provided their written informed consent to participate in this study.

## Author Contributions

CK, JP, DM, GG, and PV contributed to the design of the study. CK, GG, MT, RS, PV, and EM executed the study and analyzed the data. GG wrote the first draft of the manuscript. All authors read, revised, and approved the submitted version.

## Conflict of Interest

GG, MT, RS, DM, and JP were employed by Saluda Medical Pty Ltd. The authors declare that this study received funding from Saluda Medical. The funder had the following involvement with the study: providing the measurement device and technical personnel, financial support to the Barts Health NHS Trust to run the study.

## Publisher’s Note

All claims expressed in this article are solely those of the authors and do not necessarily represent those of their affiliated organizations, or those of the publisher, the editors and the reviewers. Any product that may be evaluated in this article, or claim that may be made by its manufacturer, is not guaranteed or endorsed by the publisher.

## References

[B1] Adelstein S. A. Lee W. Gioia K. Moskowitz D. Stamnes K. Lucioni A. (2019). Outcomes in a contemporary cohort undergoing sacral neuromodulation using optimized lead placement technique. *Neurourol. Urodyn.* 38 1595–1601. 10.1002/nau.24018 31044466

[B2] Altomare D. F. Giuratrabocchetta S. Knowles C. H. Muñoz Duyos A. Robert-Yap J. Matzel K. E. (2015). Long-term outcomes of sacral nerve stimulation for faecal incontinence. *Br. J. Surg.* 102 407–415. 10.1002/bjs.9740 25644687

[B3] Beck R. (2006). “Muscle Fiber Conduction Velocity,” in *Wiley Encyclopedia of Biomedical Engineering*, ed. Akay M. (Hoboken: John Wiley & Sons, Inc).

[B4] Brooker C. Russo M. Cousins M. J. Taylor N. Holford L. Martin R. (2021). ECAP-controlled closed-loop spinal cord stimulation efficacy and opioid reduction over 24-months: final results of the prospective, multicenter, open-label avalon study. *Pain Pract.* 21 680–691. 10.1111/papr.13008 33768664 PMC8359972

[B5] Carrington E. V. Evers J. Grossi U. Dinning P. G. Scott S. M. O’Connell P. R. (2014). A systematic review of sacral nerve stimulation mechanisms in the treatment of fecal incontinence and constipation. *Neurogastroenterol. Motil.* 26 1222–1237. 10.1111/nmo.12388 25167953

[B6] Desprez C. Damon H. Meurette G. Mege D. Faucheron J.-L. Brochard C. (2020). Ten-year evaluation of a large retrospective cohort treated by sacral nerve modulation for fecal incontinence: results of a french multicenter study. *Ann. Surg.* 10.1097/SLA.0000000000004251 [Epub Online ahead of print]. 32740249

[B7] Dumitru D. Jewett D. L. (1993). Far-field potentials. *Muscle Nerve* 16 237–254. 10.1002/mus.880160302 8446122

[B8] Dumitru D. King J. C. (1991). Far-field potentials in muscle. *Muscle Nerve* 14 981–989. 10.1002/mus.880141009 1944411

[B9] Ezra E. Siilin A. M. H. Gulobovic M. Graf J. W. R. (2020). Patterns of tined lead migration in sacral nerve modulation. *Int. J. Colorectal Dis.* 35 1163–1166. 10.1007/s00384-020-03530-0 32144532 PMC7245592

[B10] Gmel G. E. Hamilton T. J. Obradovic M. Gorman R. B. Single P. S. Chenery H. J. (2015). A new biomarker for subthalamic deep brain stimulation for patients with advanced Parkinson’s disease—a pilot study. *J. Neural Eng.* 12:066013. 10.1088/1741-2560/12/6/066013 26469805

[B11] Gourcerol G. Vitton V. Leroi A. M. Michot F. Abysique A. Bouvier M. (2011). How sacral nerve stimulation works in patients with faecal incontinence. *Colorectal Dis.* 13 e203–11. 10.1111/j.1463-1318.2011.02623.x 21689312

[B12] Hauck E. F. Schwefer M. Wittkowski W. Bothe H. W. (2009). Measurements and mapping of 282,420 nerve fibers in the S1-5 nerve roots. *J. Neurosurg. Spine* 11 255–263. 10.3171/2009.3.SPINE17684 19769506

[B13] Matzel K. E. Chartier-Kastler E. Knowles C. H. Lehur P. A. Muñoz-Duyos A. Ratto C. (2017). Sacral Neuromodulation: standardized electrode placement technique. *Neuromodulation* 20 816–824. 10.1111/ner.12695 28975677

[B14] Matzel K. E. Stadelmaie U. Gall F. P. Hohenfellner M. (1995). Electrical stimulation of sacral spinal nerves for treatment of faecal incontinence. *Lancet* 346 1124–1127. 10.1016/S0140-6736(95)91799-3 7475602

[B15] McAlees E. Vollebregt P. F. Stevens N. Dudding T. C. Emmanuel A. V. Furlong P. L. (2018). Efficacy and mechanism of sub-sensory sacral (optimised) neuromodulation in adults with faecal incontinence: study protocol for a randomised controlled trial. *Trials* 19:336. 10.1186/s13063-018-2689-1 29941019 PMC6019783

[B16] Mekhail N. Levy R. M. Deer T. R. Kapural L. Li S. Amirdelfan K. (2019). Long-term safety and efficacy of closed-loop spinal cord stimulation to treat chronic back and leg pain (Evoke): a double-blind, randomised, controlled trial. *Lancet Neurol.* 19 123–134. 10.1016/S1474-4422(19)30414-4 31870766

[B17] Norton C. Thomas L. Hill J. (2007). Management of faecal incontinence in adults: summary of NICE guidance. *BMJ* 334 1370–1371. 10.1136/bmj.39231.633275.AD 17600027 PMC1906619

[B18] Parker J. L. (2017). “Spinal Cord Stimulation for Pain Control,” in *Neuroprosthetics: Theory and Practice*, eds Horsch K. Kipke D. (Singpore: World Scientific), 710–761.

[B19] Parker J. L. Karantonis D. M. Single P. S. Obradovic M. Cousins M. J. (2012). Compound action potentials recorded in the human spinal cord during neurostimulation for pain relief. *Pain* 153 593–601. 10.1016/j.pain.2011.11.023 22188868

[B20] Parker J. L. Karantonis D. M. Single P. S. Obradovic M. Laird J. Gorman R. B. (2013). Electrically evoked compound action potentials recorded from the sheep spinal cord. *Neuromodulation* 16 295–303. 10.1111/ner.12053 23844589

[B21] Parker J. L. Shariati N. H. Karantonis D. M. (2017). Electrically evoked compound action potential recording in peripheral nerves. *Bioelectron. Med.* 1 71–83. 10.2217/bem-2017-0005

[B22] Purves D. Augustine G. J. Fitzpatrick D. Hall W. C. LaMantia A.-S. White L. E. (2012). *Neuroscience*, 5th Edn. Oxford: Oxford University Press.

[B23] Russo M. Brooker C. Cousins M. J. Taylor N. Boesel T. Sullivan R. (2020). Sustained long-term outcomes with closed-loop spinal cord stimulation: 12-month results of the prospective, multicenter, open-label avalon study. *Neurosurgery* 87 E485–E495. 10.1093/neuros/nyaa003 32023344 PMC8184296

[B24] Sandercock T. G. Faulkner J. A. Albers J. W. Abbrecht P. H. (1985). Single motor unit and fiber action potentials during fatigue. *J. Appl. Physiol.* 58 1073–1079. 10.1152/jappl.1985.58.4.1073 3988664

[B25] Thaha M. A. Abukar A. A. Thin N. N. Ramsanahie A. Knowles C. H. (2015). Sacral nerve stimulation for faecal incontinence and constipation in adults. *Cochrane Database Syst. Rev.* 8:CD004464. 10.1002/14651858.CD004464.pub3 26299888 PMC9208727

[B26] Thin N. N. Horrocks E. J. Hotouras A. Palit S. Thaha M. A. Chan C. L. H. (2013). Systematic review of the clinical effectiveness of neuromodulation in the treatment of faecal incontinence: clinical effectiveness of neuromodulation in faecal incontinence. *Br. J. Surg.* 100 1430–1447. 10.1002/bjs.9226 24037562

[B27] Vaganée D. Voorham J. Panicker J. N. Fransen E. Voorham-van der Zalm P. Van de Borne S. (2020). Neural pathway of bellows response during SNM treatment revisited: conclusive evidence for direct efferent motor response. *Neurourol. Urodyn.* 39 1576–1583. 10.1002/nau.24408 32484961

[B28] Waxman S. G. (1980). Determinants of conduction velocity in myelinated nerve fibers. *Muscle Nerve* 3 141–150. 10.1002/mus.880030207 6245357

