# Supplementary material for: Electrophysiological Responses in the Human S3 Nerve During Sacral Neuromodulation for Fecal Incontinence
Source: Front Neurosci. 2021 Oct 11;15:712168. doi: 10.3389/fnins.2021.712168 (PMC8545143; doi:10.3389/fnins.2021.712168)
Supplement: Supplementary file 1 [file Table_1.DOCX]

Supplementary Material

Supplementary table 1: Patients clinical characteristics, symptomology and results of anorectal physiologic testing.

| Patient | Sex | Age | Obstetric history^i^ | Previous surgery/events | Incontinence type | Anorectal manometry^ii^ | Rectal sensory function^iii^ | Endoanal ultrasonography^iv^ | Evacuation proctography^v^ |
| --- | --- | --- | --- | --- | --- | --- | --- | --- | --- |
| 01 | Female | 52 | 2 vaginal deliveries (1 traumatic, 1 instrumental) | Anal  Pelvic  Spinal | Urge | Normotenstion  Hypocontractility | Normal | Normal | Functional abnormality |
| 02 | Female | 76 | 2 vaginal deliveries  (1 traumatic, 1 instrumental) | Anal | Urge + Passive | Normotenstion  Hypocontractility | Normal | IAS: disrupted  EAS: atrophic | Structural abnormality |
| 03 | Male | 59 | - | Spinal (L1 injury) | Passive | Normotenstion  Hypocontractility | Hyposensitivity | Normal | - |
| 04 | Female | 64 | 2 vaginal deliveries | Pelvic  Rectal | Urge | Normotenstion  Normal contractility | Hypersensitivity | IAS: intact  EAS: disrupted | Normal |
| 05 | Female | 59 | 3 vaginal deliveries  (1 traumatic) | - | Urge + Passive | Hypotension  Hypocontractility | Normal | IAS: abnormal, focal  EAS: disrupted | Normal |
| 06 | Female | 70 | Nulliparous | Pelvic | Urge + Passive | Normal | Normal | Normal | Structural abnormality |
| 07 | Female | 52 | 2 vaginal deliveries  (2 instrumental) | Abdominal  Pelvic | Urge + Passive | Normal | Hyposensitivity | IAS: atrophic  EAS: disrupted | Functional abnormality |
| 08 | Female | 58 | 3 vaginal deliveries  (2 traumatic, 1 instrumental) | Pelvic | Urge + Passive | Normotenstion  Hypocontractility | Normal | IAS: intact  EAS: disrupted | Normal |
| 09 | Female | 47 | 4 vaginal deliveries | Pelvic  Spinal | Passive | Normotenstion  Hypocontractility | Hyposensitivity | IAS: intact  EAS: abnormal (focal), atrophic | Structural abnormality |
| 10 | Female | 65 | 2 vaginal deliveries  (1 traumatic) | - | Urge | Normotenstion  Hypocontractility | Hypersensitivity | IAS: intact  EAS: disrupted | Normal |
| 11 | Female | 74 | 5 vaginal deliveries | Anal  Pelvic  Rectal | Urge + Passive | Hypotension  Hypocontractility | Normal | IAS: atrophic EAS: disrupted | Normal |

Footnote: ^i^ Vaginal deliveries associated with an episiotomy/perineal tear were defined as traumatic; forceps/Ventouse-assisted deliveries were classified as instrumental. ^ii^ Diagnostic classification based on the London classification for disorders of anorectal function.^1^ Normal values are based on previously published work.^2 iii^ Diagnostic classification based on the London classification for disorders of anorectal function.^1^ The departmental protocol and normal values are previously described.^3 iv^ Two dimensional cross-sectional axial images of the anal canal were acquired using a 13 MHz transducer (BK Medical 2101, Berkshire, United Kingdom). Both the internal and external anal sphincter were classified as intact or abnormal (disrupted, degenerate/atrophic, or focally abnormal). ^v^ The departmental protocol is previously described.^4^ A rectal evacuation disorder was defined as being secondary to functional and/or structural abnormalities.^4^ A functional abnormality was diagnosed by incomplete (<60% instilled contrast) or protracted (>150 seconds) evacuation allied to poor opening of the anorectal angle, poor relaxation of the anal canal or poor expulsive force generated. Structural abnormalities comprised significant intussusception, rectocele, enterocele, megarectum and external rectal prolapse.^5^

**Supplementary table 2**: Motor response and myoelectric response thresholds for all analyzable experiments.

| Patient number | Stimulation electrode | Frequency [Hz] | Pulse width [μs] | Toe response threshold [mA] | Pelvic floor/anal sphincter response threshold [mA] | Myoelectric response threshold [mA] |
| --- | --- | --- | --- | --- | --- | --- |
| 01 | CH2 | 14 | 210 | 0.66 | 0.66 | 0.4 |
|  | CH3 | 14 | 210 | 3 | 2 | 1.8 |
|  | CH4 | 14 | 210 | 3.5 | 3.5 | 3 |
| 03 | CH4 | 14 | 210 | 1.9 | 1.9 | 2 |
| 05 | CH1 | 14 | 50 | - | 1.3 | 1.3 |
|  | CH2 | 14 | 50 | - | 1 | 0.99 |
|  | CH1 | 14 | 50 | - | 1.2 | 1.4 |
| 06 | CH1 | 14 | 210 | 0.4 | 0.2 | 0.5 |
|  | CH2 | 14 | 210 | 0.4 | 0.2 | 0.6 |
|  | CH3 | 14 | 210 | 0.45 | 0.35 | 0.3 |
|  | CH4 | 14 | 210 | not reported | 0.45 | 0.46 |
|  | CH4 | 14 | 30 | 1.9 | 1.9 | 1.8 |
| 07 | CH1 | 14 | 210 | not reported | 0.9 | 0.5 |
|  | CH2 | 14 | 210 | 1.2 | 0.8 | 0.6 |
|  | CH3 | 14 | 210 | - | 0.4 | 0.39 |
|  | CH4 | 14 | 210 | not reported | 0.3 | 0.3 |
| 09 | CH1 | 5 | 210 | 2 | 1 | 0.6 |
|  | CH2 | 14 | 210 | 1.7 | 1 | 0.7 |
|  | CH3 | 14 | 210 | 2 | 1 | 0.6 |
| 10 | CH1 | 14 | 210 | not reported | 1.6 | 1 |
|  | CH2 | 14 | 210 | - | 1.6 | 1.6 |
|  | CH3 | 14 | 210 | not reported | 2.5 | 2.5 |
|  | CH4 | 14 | 210 | not reported | 1.4 | 1.4 |

**Footnote:** For toe responses, ‘-‘ denotes that no toe response was observed. The label ‘not reported’ indicates that the focus of the team in the operating theater was on the pelvic floor/anal sphincter response and the toe response was not monitored or reported accurately on an individual level.

**Supplementary material references**

1. Carrington EV, Heinrich H, Knowles CH, et al. The international anorectal physiology working group (IAPWG) recommendations: Standardized testing protocol and the London classification for disorders of anorectal function. Neurogastroenterol Motil 2019:e13679.
2. Carrington EV, Brokjaer A, Craven H, et al. Traditional measures of normal anal sphincter function using high-resolution anorectal manometry (HRAM) in 115 healthy volunteers. Neurogastroenterol Motil 2014;26:625-35.
3. Townsend DC, Carrington EV, Grossi U, et al. Pathophysiology of fecal incontinence differs between men and women: a case-matched study in 200 patients. Neurogastroenterol Motil 2016;28:1580-8.
4. Palit S, Bhan C, Lunniss PJ, et al. Evacuation proctography: a reappraisal of normal variability. Colorectal Dis 2014;16:538-46.
5. Grossi U, Di Tanna GL, Heinrich H, et al. Systematic review with meta-analysis: defecography should be a first-line diagnostic modality in patients with refractory constipation. Aliment Pharmacol Ther 2018;48:1186-1201.
